# Speech Prosody Serves Temporal Prediction of Language via Contextual Entrainment

**DOI:** 10.1523/JNEUROSCI.1041-23.2024

**Published:** 2024-06-05

**Authors:** Yulia Lamekina, Lorenzo Titone, Burkhard Maess, Lars Meyer

**Affiliations:** ^1^Research Group Language Cycles, Max Planck Institute for Human Cognitive and Brain Sciences, Leipzig 04103, Germany; ^2^Methods and Development Group Brain Networks, Max Planck Institute for Human Cognitive and Brain Sciences, Leipzig 04103, Germany; ^3^University Clinic Münster, Münster 48149, Germany

**Keywords:** language comprehension, neural entrainment, prosody, temporal predictions

## Abstract

Temporal prediction assists language comprehension. In a series of recent behavioral studies, we have shown that listeners specifically employ rhythmic modulations of prosody to estimate the duration of upcoming sentences, thereby speeding up comprehension. In the current human magnetoencephalography (MEG) study on participants of either sex, we show that the human brain achieves this function through a mechanism termed entrainment. Through entrainment, electrophysiological brain activity maintains and continues contextual rhythms beyond their offset. Our experiment combined exposure to repetitive prosodic contours with the subsequent presentation of visual sentences that either matched or mismatched the duration of the preceding contour. During exposure to prosodic contours, we observed MEG coherence with the contours, which was source-localized to right-hemispheric auditory areas. During the processing of the visual targets, activity at the frequency of the preceding contour was still detectable in the MEG; yet sources shifted to the (left) frontal cortex, in line with a functional inheritance of the rhythmic acoustic context for prediction. Strikingly, when the target sentence was shorter than expected from the preceding contour, an omission response appeared in the evoked potential record. We conclude that prosodic entrainment is a functional mechanism of temporal prediction in language comprehension. In general, acoustic rhythms appear to endow language for employing the brain's electrophysiological mechanisms of temporal prediction.

## Significance Statement

Language comprehension benefits from our ability to predict upcoming stimuli. Here, we report on a key neural substrate. We show that electrophysiological brain activity inherits prosodic modulations—the melody of speech—from prior context, allowing listeners to estimate the duration of upcoming language stimuli. By using magnetoencephalography, we find that the brain not only responds to prosody when speech is present, but its activity continues at the prosodic frequency seconds into the future, benefiting behavioral responses. During continuation, activity shifts from the auditory to the frontal cortex, the epicenter of the brain's predictive abilities. The human brain seems to initiate the top-down prediction of language stimuli by copying sensory rhythms and projecting them into the future.

## Introduction

Predicting upcoming linguistic information allows us to process language quickly and efficiently (Sohoglu et al., 2012; Li and Zhang, 2023). Predictions are generated across different levels of linguistic content, including phonology (DeLong et al., 2005), syntax (Van Berkum et al., 2005; Lau et al., 2016), and (lexical) semantics (Demberg and Keller, 2008; Boston et al., 2011; Smith and Levy, 2013).

In addition to content, listeners predict the timing of upcoming linguistic units. On slow time scales, listeners employ speech prosody to infer the duration of upcoming sentence segments (Grosjean, 1983). Cues such as pauses, pitch modulations, and duration changes trigger the bottom-up segmentation of sentences into syntactic phrases (Frazier et al., 2006). Crucially, the interpretation of prosodic cues depends on a wider prosodic context. For instance, intonational phrase boundaries (IPBs) are not processed in terms of their absolute acoustic magnitude but relative to the magnitude of preceding IPBs (Clifton et al., 2002; Snedeker and Casserly, 2010). Moreover, even distant prosodic cues can influence the prediction of upcoming material, such that prosody at sentence onset affects subsequent segmentation and word recognition (Dilley and McAuley, 2008; Brown et al., 2011). Furthermore, during reading, where acoustic cues are unavailable, listeners actively construct implicit prosody that facilitates speech processing (Fodor, 2002; Breen, 2014; Breen et al., 2016a). Consequently, context effects have also been reported across perceptual modalities. In previous studies, it was observed that prosodic contours can trigger downstream effects that persist beyond stimulation, affecting the comprehension of upcoming visual sentences devoid of prosody (Steinhauer and Friederici, 2001).

Recent behavioral findings suggest that temporal prediction, including the effects of prosody, may be triggered by rhythmic or repetitive contexts. For instance, rhythmic amplitude-modulated sounds at a given frequency induce perceptual detectability of subsequent targets that arrive at the same frequency (Hickok et al., 2015). Furthermore, the prosodic syllable rate of a lead-in sentence can affect the detection of subsequent target syllables. After a fast-rate sentence, subjects overhear short target syllables that they do perceive when the lead-in sentence is presented at a slow rate ( Dilley and Pitt, 2010; Bosker, 2017). Corpus evidence further suggests that language prosody exhibits sufficient rhythmicity for temporal predictions to emerge (Inbar et al., 2020; Stehwien and Meyer, 2021).

In a recent set of behavioral studies, we found supporting evidence that the facilitatory effect of prosodic context on temporal prediction hinges specifically on the rhythm of prosody (Lamekina and Meyer, 2022). We had participants listen to repetitive prosodic contours, followed by visual target sentences that either matched or not in duration. We showed that a duration match accelerated the processing of the target sentence, indicating that listeners predict sentence duration.

In the current study, utilizing the same paradigm in a magnetoencephalography (MEG) experiment, we report that the behavioral prediction benefit of rhythmic prosodic contexts is driven by brain activity that resonates with such rhythm. Prior work has reported narrow-band electrophysiological activity to tune to rhythmic stimuli and thereby optimize the processing of upcoming input (Lakatos et al., 2008; Schroeder and Lakatos, 2009; Henry and Obleser, 2012). Specifically, activity in the delta range (<4 Hz) triggers auditory temporal duration predictions through an electrophysiological mechanism termed entrainment, by which the brain inherits a stimulation frequency to persist after stimulus offset (Stefanics et al., 2010; Breska and Deouell, 2017; Herbst et al., 2022). This mechanism could also potentially affect the processing of upcoming language stimuli. In the domain of speech and language, delta-band activity is known to synchronize with prosody (Luo and Poeppel, 2007; Bourguignon et al., 2013), but predictive functions have not been reported.

**Figure 1.**
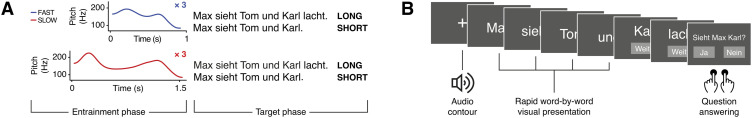
Paradigm and procedure. ***A***, Paradigm. Prosodic contour repeated three times to induce entrainment, followed by time-matched rapid serial visual presentation of the target sentence. FAST entrainment facilitates comprehension of SHORT sentences, and SLOW entrainment facilitates comprehension of LONG sentences (Lamekina and Meyer, 2022). ***B***, Procedure. The participants first listened to the audio contour. Then the sentence was presented word by word. In 75% of the trials, a comprehension question followed.

To investigate whether delta-band prosodic entrainment subserves sustained temporal predictions during subsequent sentence processing, we designed an MEG study that combined an initial repetitive prosodic rhythm (entrainment phase) with a subsequent visual sentence presentation (target phase; Fig. 1*A*). The visual modality for the target phase was chosen in order to avoid confounding prosodic characteristics in auditory target stimuli. Similar cross-modal paradigms have been successfully implemented in previous studies (Steinhauer and Friederici, 2001), which showed that a stable effect is also obtained across modalities. Our experiment employed prosodic contours that either matched or mismatched the duration of a subsequent visual target sentence. Our behavioral results (Lamekina and Meyer, 2022) showed that a duration match is indeed associated with a behavioral benefit. On the neural level, we expected this effect to emerge due to rhythmic prosodic entrainment. That is, we first hypothesized that delta-band activity would synchronize with the modulation frequency of the contours. Second, we expected that brain activity at the modulation frequency would still be detectable during the processing of the visual target sentence. Third, we expected an omission event-related field (ERF) response when the duration of the target sentence mismatched the duration of the entraining contours (Scharinger et al., 2017; Ragazzoni et al., 2019).

## Materials and Methods

### Participants

We conducted the experiment on 40 participants (German native speakers; right-handed; 19 females; age range, 18–35 years; mean age = 28 years; SD = 4 years). The MEG data was recorded from different participants and in a separate session from the preceding behavioral study with a similar paradigm (Lamekina and Meyer, 2022). Five participants were further excluded from the analysis due to noise in the MEG; the resulting sample was 35 subjects. The participants had normal or corrected-to-normal vision and no reported history of neurological or hearing disorders. Participation was reimbursed with € 12 per hour. All subjects were naive to the purpose of the study. Written informed consent was obtained prior to the experiment. The study conformed to the guidelines of the Declaration of Helsinki and was approved by the local ethics committee of the University of Leipzig, Germany (approval no. 060/17-ek).

### Experimental design and statistical analysis

#### Stimuli and paradigm

In order to investigate the potential influence of prosodic entrainment on subsequent sentence comprehension, the trials of our experiment combined an initial prosodic rhythm with a subsequent visual target sentence. This prosodic contour always belonged to one of the conditions—SLOW or FAST—characterized by different lengths. Importantly, although the main manipulation for the contours was the duration change, the contours were repeated three times, resulting in three cycles with a period different for every condition. This additional manipulation was introduced in order to induce stimuli rhythmicity and an electrophysiological brain response at the same frequency. Contour repetitions had pauses of 160 ms in between. This duration of the pause has previously been shown to be sufficient in eliciting entrainment to prosodic stimuli (Ghitza, 2017). Together with the pause, one cycle of SLOW contour lasted for 1.73 s, corresponding to a frequency of approximately 0.6 Hz. One cycle of the FAST contour together with the pause lasted for 1.1 s, corresponding to 0.9 Hz. An additional pause matching the difference in duration between the FAST and SLOW contours was added before the FAST contour to equalize duration across conditions.

Prosodic contour exposure was followed by a visual word-by-word presentation of a target sentence. Critically, the presentation of the visual words was adjusted to contour duration, whereby the word presentation rate was calculated from the same auditory sentences from which the contour was extracted. Target visual sentences were either LONG or SHORT. The LONG sentences had an exact duration of 1.884 s (314 ms × 6 words), and the SHORT sentences had an exact length of 1.57 s (314 ms × 5 words). The presentation of sentences, therefore, corresponded in timing either to the SLOW contour without pause (five words) or to the FAST contour without pause (6 = 3 + 3 words; compare Fig. 1).

After a sentence presentation and a delay, some trials were followed by comprehension questions (see below, Procedure). Stimuli and procedure were adapted from Experiment 1 in our previous behavioral study on prosodic entrainment (Lamekina and Meyer, 2022). Therefore, in the following paragraphs, we reiterate the description of the stimulus material used in the previous study.

For examples of target sentences, see below:

(1). LONG: Max sieht Tom und Karl lacht.
  “Max sees Tom and Karl laughs.”
(2). SHORT: Max sieht Tom und Karl.
  “Max sees Tom and Karl.”

For sentence construction, we used 32 monosyllabic first names of 3–6 characters to balance the word-by-word presentation (New et al., 2006). Noun frequencies were normally distributed (Heister et al., 2011). Since male and female first names differ in length, we used male first names only. We also selected 75 transitive and 75 intransitive German verbs in the third-person singular present tense. The length was matched (1–2 syllables, 5–8 characters). Verb frequencies were also normally distributed. Pairs of transitive and intransitive verbs were made based on semantic fit (e.g., expect–come, wake up–sleep). A combination of verb pairs and names yielded 6,000 sentences. A different name triplet was used for each of these. Name triplets were selected to not contain similar-sounding names (e.g., Frank and Franz).

Prosodic contours of two different rates were presented before the target sentences. Contours were made by averaging the pitch tracks of the visual sentences, which were stripped off from synthetic recordings (Oord et al., 2016) in Praat (Boersma and van Heuven, 2001). We used a female voice (minimum pitch, 116 Hz; maximum pitch, 267 Hz; average pitch, 191.5 Hz) for its broad pitch range and high variability. The two entrainment rates were SLOW (based on the 3,000 five-word SHORT sentences; e.g., Max sieht Tom und Karl) and FAST, based on 3,000 additional partial three-word sentences (e.g., Max sieht Tom).

For averaging, contour durations were adjusted to the average duration of the respective sentence recordings (SLOW, 1,570 ms; FAST, 942 ms). The contours within each condition had an identical duration in order to increase the signal-to-noise ratio (SNR). For the purpose of our experiment, the contours needed to be delexicalized, i.e., stripped off any lexical content/acoustic fine structure, so that only prosodic characteristics remain. To achieve this aim, the Prosody Unveiling through Restricted Representation (PURR) method was used (Sonntag and Portele, 1998). This pipeline was recommended in previous studies for constructing a delexicalized prosodic contour (Steinhauer and Friederici, 2001). The method involves extracting the pitch values from the original contours and constructing a sound by adding a sine wave at a pitch, its second harmonic of ¼ of the amplitude, and its third harmonic of 1/16 of the amplitude (suggested by Klasmeyer, 1997). Therefore, out of the original spectral characteristics of the speech signal, only the pitch modulations are retained, which permits to disentangling of prosodic modulations from other speech components. The prosodic pitch has proved to provide a substantial contribution to entrainment, separately from other acoustic and phonetic features (Teoh et al., 2019). PURR has been tested extensively and compared with other methods, proving over a variety of experiments to have the best functionality and acceptability (listeners recognizing the signal as coming from natural human speech) for speech delexicalisation (M. Meyer et al., 2002; Kotz et al., 2003; Pannekamp et al., 2005). Contours were further normalized to 65 dB and lowered in pitch by 55 Hz to ensure a comfortable hearing level. The average word duration for timed visual presentation calculated from the synthesized contour length was 314 ms.

The 6,000 sentences and contours were combined into 20 experimental lists of 300 trials each. Within the list, every verb pair was used four times, once within each condition (i.e., SHORT–FAST, SHORT–SLOW, LONG–FAST, and LONG–SLOW). Pairs and conditions did not repeat across subsequent trials. We disallowed adjacent name triplets with identical or similar names. Identical triplets did not repeat within the list.

#### Procedure

Each trial started with a visual fixation cross and auditory presentation of one of the two prosodic contours, repeated three times (Fig. 1*B*). After a contour, a target sentence (either SHORT or LONG) was presented word by word. The words were shown in a rapid serial visual presentation (RSVP; Young, 1984). Word presentation rate was identical across conditions (314 ms/word); critically, it was adjusted to contour duration to allow for assessing prosodic entrainment. In contrast to our behavioral study (Lamekina and Meyer, 2022), we adapted the procedure to better suit the purposes of the current experiment: the final words of the sentence were no longer self-paced, that is, RSVP was used for all words of the sentence. This was done to avoid muscle artifacts associated with the button presses. It would also be worthwhile to note that single words could induce their own neural responses in the target phase at a frequency of ∼3 Hz; however, this doesn't match our frequencies of interest (0.6 and 0.9 Hz) and, in our view, presents no obstacles or interest for further analysis.

After visual sentence presentation and a jittered delay of 500+ 0–250 ms, comprehension questions were presented in 75% of the trials. To avoid strategy buildup, questions requiring a “yes” and a “no” answer were both included for each condition (see Lamekina and Meyer, 2022 for details). Both entrainment conditions were matched on the types of questions and the amount of correct “yes” and “no” answers. The “yes/no” button assignment was matched across the participants. There was a response timeout of 2,000 ms. In case of timeout, a screen stating “Please answer faster” appeared, and the experiment advanced to the next trial. The participants were instructed to listen to the audio contour, read the sentences presented word by word, and then answer a comprehension question after some trials. The 300 trials of the experiment ran in five blocks, with self-regulated pauses in between (every block took approximately 10 min). The whole experiment lasted approximately 60–70 min, depending on the duration of the pauses.

#### Data recording and preprocessing

##### MEG recording

The experiment was conducted in an electromagnetically shielded room in a single session. Stimuli were presented using the Presentation software (Neurobehavioral Systems). The participants heard the prosodic contours through air-conduction earplugs (ER3-14A/B, Etymotic Research) connected via a 50 cm plastic tube to piezo phones (TIP-300, Nicolet Biomedical). Visual stimuli were back-projected on a semitransparent screen from a projector located outside of the magnetic shielding room (Panasonic PT-D7700E, Matsushita Electric Industrial). The screen was located ∼90 cm from each participant. MEG signals were measured at a sampling rate of 1,000 Hz within a passband of 0–330 Hz from 306 sensors, including 102 magnetometers and 204 gradiometers (Vectorview, Elekta Neuromag Oy). The head position inside the helmet was continuously monitored using five head-tracking coils.

##### MEG preprocessing

Each block of the data was separately corrected for head movements and external noise with the signal space separation (SSS) method (Taulu et al., 2005) via the MaxFilter software (MaxFilter Version 2.2.15, Elekta Oy) utilizing spherical functions up to eleventh order for the head field model and up to the second order for the environmental field model. Movements were compensated using a 500 ms window while forwarding with steps of 250 ms. The temporal correction was computed using a 10 s time window; correlations higher than 0.98 between inside and outside field components were projected out. Line frequency was set to 50 Hz. Further analyses were conducted in MATLAB (The MathWorks) via the FieldTrip toolbox (Oostenveld et al., 2011). Since previous research has shown that results obtained from different types of MEG sensors after SSS are highly correlated (Garcés et al., 2017), we restricted the analysis to magnetometers only. The data were first low-pass filtered at 90 Hz [one-pass, zero-phase–shift finite impulse response filter with windowed sinc, Kaiser window], detrended, and demeaned. Line noise (50 Hz) and its harmonics were removed from the signal using ZapLine (de Cheveigné, 2020). Noisy channels were identified semiautomatically: Candidates in every block were chosen when the maximum absolute value in the channel exceeded a threshold of 6 pT; those candidates were further confirmed by visual inspection. We excluded five participants who had >10% of noisy channels; all further analyses were conducted on 35 subjects. In this final sample, the mean number of removed channels per subject was 2 out of 102 channels (SD = 2). After this procedure, the data were downsampled to 250 Hz and segmented into epochs, each comprising one contour and the following visual sentence (ranging from 6,480 to 8,530 ms depending on the four combinations of prosodic and sentence conditions). For coherence, power, and ERF analyses, smaller segments were cut out separately at later analysis stages (see below). Epoched data were further subjected to artifact rejection. First, trials containing SQUID jump artifacts were detected using *z*-transformation of the data at each time point. These *z*-values were calculated from the ninth-order median-filtered data. The *z*-value threshold of the jump detection was set to 75; trials with *z*-values exceeding this threshold were excluded. A similar method was used for the automatic detection of trials with muscle artifacts (frontotemporal channels; prior bandpass filtering from 110 to 124 Hz; *z*-value threshold - 20). For this step, trials were zero-padded prior to filtering to avoid edge artifacts. Finally, trials that contained time points with values exceeding ±6 pT were excluded. The whole artifact detection procedure led to the removal of a mean of four trials per block (SD = 4). The preprocessed data were further subjected to independent component analysis to remove ocular and cardiac artifacts. Artifact components were identified based on visual inspection of component waveform and topography. The MEG data were then reconstructed in the original sensor space, excluding the artifactual components. Excluded channels were then interpolated within the block using spline interpolation and a template neighborhood structure for Elekta Neuromag Oy magnetometers. Finally, the preprocessed data from the five blocks per subject were concatenated. Further analyses in sensor space were conducted on the subject level. In the source space, we ran the analysis on the block level due to differences in MEG sensor positions and, therefore, different lead fields for every recording block.

##### MRI preprocessing

In order to identify neural sources underlying the prosodic entrainment mechanism, anatomically constrained source localization was used. First, individual T1-weighted MRI images obtained with a 3T MRI scanner (Magnetom Trio, Siemens AG) were segmented using the FreeSurfer software (http://surfer.nmr.mgh.harvard.edu/). For every participant, MEG data were coregistered with the individual MRI scan via realignment of the fiducial (nasion, left, and right preauricular) and digitized head surface points (acquired with Polhemus FASTRAK 3D digitizer). To this end, we used a semiautomated iterative procedure (Besl and McKay, 1992) implemented in the MNE software (Massachusetts General Hospital; http://www.nmr.mgh.harvard.edu/martinos/userInfo/data/). Next, using MNE, volume conductors were constructed as boundary element models, resulting in individual inner-skull surfaces comprising 2,562 vertices each. Based on the volume conductors, individual head models were created in FieldTrip using the single-shell model (Nolte, 2003). In parallel, source spaces consisting of 10,242 vertices per hemisphere were constructed in MNE. To arrive at results that are comparable between subjects in terms of neuroanatomical function and structure, every subject's source space was parcellated into the regions of the HCPMMP1 atlas (Glasser et al., 2016); all source space analyses were carried out on atlas-defined regions (Fischl et al., 1999). Based on the individual head models, source spaces, and MEG sensor positions in the current recording block, separate lead fields for every subject and block were calculated in FieldTrip. Lead fields were normalized in order to remove depth bias (Van Veen et al., 1997).

#### Sensor space analysis

##### Frequency data extraction

Sensor space analyses were performed on separate epochs for the entrainment and target phases to avoid spectral leakage affecting the results. First, in order to assess neural tracking of speech prosody at delta-band frequency in the entrainment phase, we calculated pitch–MEG coherence at 0.6 Hz (rate of the SLOW contour) and 0.9 Hz (rate of the FAST contour). For this analysis, we cut out epochs starting from contour onset and corresponding to the duration of entrainment (5.19 s for SLOW and 3.32 s for FAST). All epochs had a high signal-to-noise ratio (SNR > 2). The *F*_0_ envelope was calculated using Praat (Boersma and van Heuven, 2001). Since *F*_0_ is undefined for pauses, those were interpolated using spline interpolation. Both the speech envelope and the MEG data underwent a fast Fourier transform (FFT) with a Hann window.

**Figure 2.**
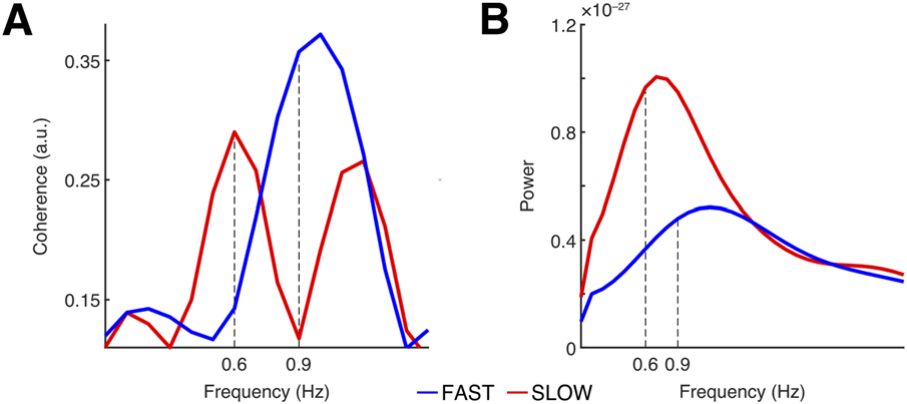
Sensor-level spectra. ***A***, Coherence spectrum in the entrainment phase. Distinct peaks are visible: 0.9 Hz for the FAST condition and 0.6 and 1.2 (harmonics of 0.6) for the SLOW condition. These peaks correspond to the respective occurrence frequencies of the contours. The *x*-axis is limited to 1.5 Hz for display purposes. ***B***, Power spectrum in the target phase. Peaks visible around frequencies of interest: 0.6  and 0.9 Hz for the SLOW and FAST conditions, respectively. The *x*-axis is adjusted to 3 Hz for display purposes.

Since pitch–MEG coherence values were no longer accessible in the target phase (due to the acoustic stimulus not being present), we followed our hypothesis of sustained entrainment by quantifying power at the two frequencies of interest for both conditions in this phase. To this end, we segmented the data into epochs starting from visual sentence onset and corresponding in duration to a single prosodic contour (1.73 s for SLOW and 1.10 s for FAST). These time windows were chosen in accordance with the hypothesis that cortical entrainment is sustained for the duration of at least one period at the respective frequency. Data from these epochs were also subjected to a fast Fourier transform with a Hann window. For both phases, FFT was restricted to a frequency range of 0–3 Hz. Because frequency comparison between 0.6 and 0.9 Hz required a resolution of 0.1 Hz, we zero-padded each trial from every phase up to a duration of 10 s. Coherence (entrainment phase) and power (target phase) were then calculated for 31 bins with a step size of 0.1 Hz (Rosenberg et al., 1989). Visual inspection suggested that both the coherence and power spectra for the SLOW contour exhibited a peak at 0.6 Hz (the coherence spectrum also showed a peak at 1.2 Hz, which is the harmonics of 0.6 Hz), while the spectra for the FAST contour peaked at 0.9 Hz (Fig. 2). The power spectrum in sensor space, however, could have been obscured by the presence of a 3 Hz rhythm induced by visual words (see above, Procedure); yet the peaks around the target frequencies are still clearly visible. The peaks become more clearly defined in the spectrum on the source level (see below, Source space analysis).

##### Statistical frequency analysis

Statistical analysis for coherence and power was conducted on the two frequencies of interest (0.6 and 0.9 Hz) using nonparametric cluster-based permutation tests (Maris and Oostenveld, 2007), with 5,000 permutations (*α* = 0.05, ≥3 channels minimum cluster size). We used a template neighborhood structure for Elekta Neuromag Oy magnetometers. Because implementation of a two-way test is not straightforward in the cluster permutation framework, we instead conducted multiple two-tailed whole-brain paired-samples *t* tests: 0.6 versus 0.9 Hz in SLOW, 0.6 versus 0.9 Hz in FAST, and a comparison of the difference 0.6 versus 0.9 Hz in SLOW to the difference 0.6 versus 0.9 Hz in FAST.

##### ERF data extraction

In order to investigate our hypothesis on the omission ERF in SHORT sentences after SLOW contours, we analyzed only the SHORT sentences at the onset of the missing verb (which was never presented) as a function of the two conditions of prior prosodic contour—SLOW or FAST; that is, the physical stimulus that was present during the measured ERFs was identical (i.e., no stimulus/omission), only the prior prosodic context differed. Therefore, the ERF omission effect could not be attributed to differences in the visual sentences themselves (as we used identical sentences) but rather to the differences in prior prosodic conditions. To this aim, we epoched the data from SHORT sentences in both prosodic conditions around the theoretical onset of the verb (−250 ms preonset, 1 s postonset). Baseline correction was applied, demeaning the waveforms with the 250 ms interval preceding the onset of the verb. All epochs had a high signal-to-noise ratio (SNR > 2). ERFs were calculated within the subject and within the FAST and SLOW conditions and subjected to statistical analysis.

##### Statistical ERF analysis

For statistical analysis, we employed a cluster-based permutation test on the time interval from 300 to 600 ms after the onset of the missing verb (5,000 permutations, *α* = 0.05, ≥3 channels minimum cluster size). The time window was chosen based on the previous literature regarding the timing of omission response (Penney, 2004; Scharinger et al., 2017; Ragazzoni et al., 2019), as well as on a general consideration that omission response can bear characteristics of both P300 and P600 components (Nakano et al., 2014; cf. Sassenhagen and Fiebach, 2019).

#### Source space analysis

##### Frequency data extraction

Source space analyses of coherence during the entrainment phase and power during the target phase were performed within block; reconstructions were averaged across blocks. To estimate source-level tracking of prosody in the entrainment phase, cross-spectral density (CSD) matrices were computed for all combinations of MEG channels and the pitch track using fast Fourier transforms (FFT) with Hann tapering; data were zero-padded to 20 s to achieve sufficient frequency resolution. To source-localize individual conditions while avoiding single-condition bias (Gross et al., 2001; van Vliet et al., 2018), we employed common spatial filters. First, CSD matrices were calculated separately for each frequency of interest (0.6/0.9 Hz) but common to the SLOW and FAST conditions. Based on the two CSD matrices, two spatial filters were then constructed using dynamic imaging of coherent sources (Gross et al., 2001) within the subject's volume conductor and source space. To source-localize the individual conditions, we then calculated individual CSD matrices for each of the four frequency–condition combinations in the entrainment phase. Next, we applied each of the two spatial filters (0.6/0.9 Hz) to the SLOW and FAST CSD matrices within that filter's frequency. To additionally localize the sustained entrainment effect from the sensor space power analysis, source analysis of power in the target phase was conducted using an analogous pipeline.

Subsequently, single-node coherence values (entrainment phase) and power values (target phase) obtained from the beamformer analyses for every frequency and condition were averaged across each area provided by the individual atlas parcellation (360 areas, 180 per hemisphere). Finally, the values were averaged across blocks for every participant and subjected to statistical analysis on the a priori-defined regions of interest (ROI).

The ROI pattern comprised four regions, based on previous literature. Firstly, we looked at the superior temporal gyrus (STG), known for its involvement in prosodic (Bourguignon et al., 2013; M. Meyer et al., 2002, 2004; Sammler et al., 2015) as well as general linguistic (Friederici et al., 2003; Vigneau et al., 2006; Friederici, 2011) processing. Secondly, we focused on the early auditory cortex (EAC or Heschl's gyrus), which is responsible for general acoustic processing and is known to synchronize with speech prosody (M. Meyer et al., 2002, 2004). Thirdly, we investigated the inferior frontal gyrus (IFG), closely related to general linguistic (Friederici et al., 2006; Goucha and Friederici, 2015; Zaccarella and Friederici, 2015; Van der Burght et al., 2021 etc.), prosodic (Frühholz and Grandjean, 2013; Sammler et al., 2015), and predictive (Jakuszeit et al., 2013; Matchin et al., 2017; Avenanti et al., 2018) processing. Finally, we looked at frontal operculum (FOP), also known to be involved in prosodic (Kotz et al., 2003; M. Meyer et al., 2002, 2004) and predictive (Bubic et al., 2009) processing. For the complete list of the atlas subareas that were combined into each respective ROI, please refer to Extended Data Table 3-1.

For visual inspection of the coherence and power spectra in order to confirm the corresponding peaks at the frequencies of interest, we additionally reiterated the same source analysis procedure for each frequency in a limited spectrum of 0–3 Hz with a step of 0.1 Hz.

##### Statistical frequency analysis

To investigate statistical differences among frequencies and conditions within the ROIs, we used a series of linear mixed-effects (LME) regression models, implemented in R (R Core Team, 2021) in the lme4 package (Bates et al., 2015). The source reconstruction data was averaged across the abovementioned ROIs and split by hemispheres. Two experimental phases were analyzed separately. First, to investigate the entrainment effect in every phase, we ran separate models on every ROI with CONDITION (SLOW vs FAST) and FREQUENCY (0.6 vs 0.9) and their interaction as predictors. The dependent variables were coherence (entrainment phase) and power (target phase). Since the entrainment effect proved to be significant in all ROIs (see below in Results), we further proceeded to estimate in which area/hemisphere the effect was statistically higher. To this end, we required a measure that permits to quantify the overall strength of entrainment in a particular region. For this purpose, we adapted an index termed “rate-specific response” (RSR) from previous literature (RSR; van Bree et al., 2021). This index is calculated as follows:

$$\mathrm{RSR}=(R_{0.6,\;\mathrm{SLOW}}-R_{0.6,\;\mathrm{FAST}})+(R_{0.9,\;\mathrm{FAST}}-R_{0.9,\;\mathrm{SLOW}}),$$

where *R* is the response measure (coherence or power) and 0.6/0.9 Hz and SLOW/FAST are, respectively, the frequencies of interest and conditions, for which this response measure is calculated. An RSR is larger than 0 when electrophysiological brain activity follows the initial speech rate for both conditions (stronger 0.6 Hz coherence/power for the SLOW condition and stronger 0.9 Hz coherence/power for the FAST condition). That is, higher RSR indicates a brain response that is specific, or entrained, to the stimulus rate. We subjected the resulting RSR indices for every area and participant to new linear mixed-effects regression models (a separate model for every phase), where the predictors were now AREA, HEMISPHERE, and their interaction. The dependent variables were now corresponding RSR indices, reflecting the entrainment strength.

For all models, predictors were coded using mean-centered effects coding. Random intercepts were included for subjects. The models were further subjected to an ANOVA analysis in order to determine the significance of the predictors.

##### ERF data extraction

To source-localize the omission ERF effect, we used the linear constrained minimum variance beamformer (Van Veen et al., 1997). As the omission can only be studied for SHORT sentences, we first constructed a covariance matrix for the SHORT sentences from both entrainment conditions in the ERF time window (−250 to 1,000 ms around the potential onset of the critical word; baseline corrected). This matrix, together with individual volume conductors and lead fields, was used to compute the common spatial filter for every block of data. Next, source reconstruction was performed separately for every condition using the precomputed common filter and the individual covariance matrix corresponding to the condition. The resulting single-voxel time courses were averaged across every atlas area and further averaged across blocks for every subject. From these subject-level time courses, we then selected and averaged data across the time window of interest (300–600 ms).

##### Statistical ERF analysis

Since we did not have a strict predefined hypothesis for the source localization of the omission ERF, we did not preselect any ROIs. Instead, for every atlas area, we performed a two-tailed paired-sample *t* test in order to determine the differences between SLOW and FAST entrainment conditions (*α* = 0.05). For illustration, we report the ten regions across hemispheres where the difference between conditions was maximally significant (*p*-values uncorrected).

## Results

### Delta-band activity synchronizes with speech prosody

#### Sensor space – frequency analysis

**Figure 3.**
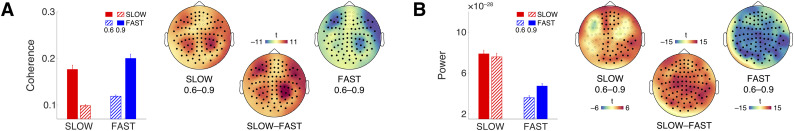
Sensor space results. ***A***, Entrainment phase. Left: Coherence values, averaged over participants and sensors (mean ± standard error). Coherence at 0.6 Hz is higher in the SLOW condition, while at 0.9 Hz, it is higher in the FAST condition. Right: Topographic distribution of coherence *T*-values of the cluster permutation tests. ***B***, Target phase. Left: Power values, averaged over participants and sensors (mean ± standard error). Power at 0.6 Hz is higher in the SLOW condition, while at 0.9 Hz, it is higher in the FAST condition. Right: Topographic distribution of power *T*-values of the cluster permutation tests (scales adjusted for demonstrative purposes). For the MEG sensors layout, see Extended Data Figure 2-1.

During the entrainment phase, analysis for the 0.6 versus 0.9 Hz contrast in SLOW revealed a significant positive cluster (cluster-sum *t*_(34)_ = 258, cluster-level *p* < 0.001, corrected; peak at sensor MEG2441, peak-level *t*_(34)_ = 10, peak-level *p* < 0.001; for MEG sensors layout, see Extended Data Fig. 2-1). This cluster comprised all but six sensors. In turn, the 0.6 versus 0.9 Hz contrast in FAST revealed a significant negative cluster (cluster-sum *t*_(34)_ = −291, cluster-level *p* < 0.001, corrected; peak at sensor MEG1421, peak-level *t*_(34)_ = −11.91, peak-level *p* < 0.001). This cluster also included all but nine sensors. Finally, the comparison of difference maps (i.e., SLOW vs FAST) yielded a significant positive cluster including all but one sensors (cluster-sum *t*_(34)_ = 396, cluster-level *p* < 0.001, corrected; peak at sensor MEG2221, peak-level *t*_(34)_ = 12.33, peak-level *p* < 0.001). Peak sensors for SLOW and difference clusters were located in the right parietal region, while the peak sensor for the FAST cluster was located in the right frontotemporal region. Overall, these results suggest that brain activity synchronizes with the rate of the external prosodic contour (Fig. 3*A*).

10.1523/JNEUROSCI.1041-23.2024.f2-1 Figure 2-1 Download Figure 2-1, TIF file.

#### Source space – frequency analysis

**Figure 4.**
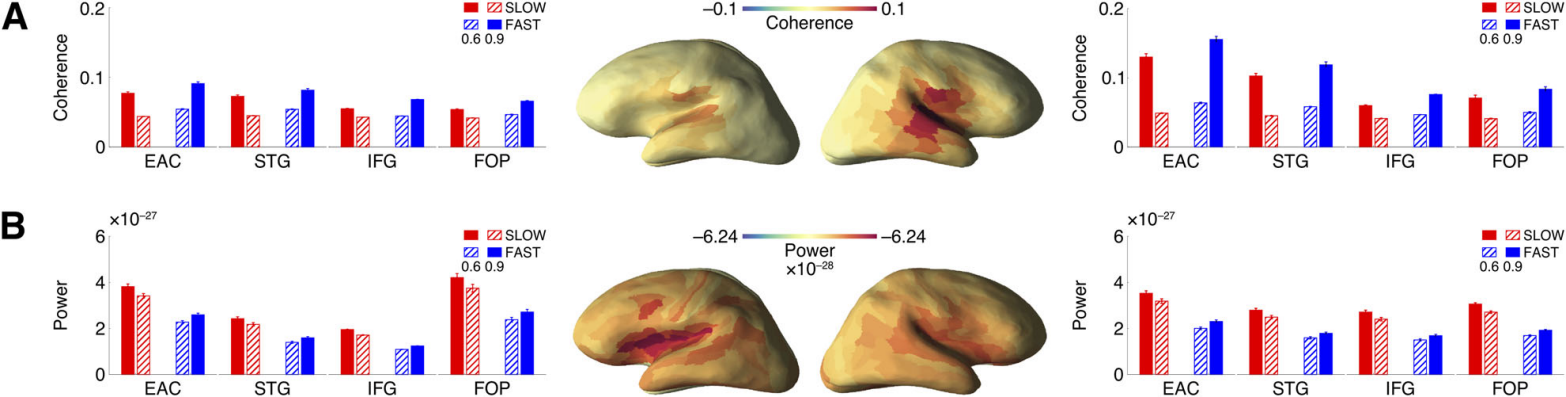
Source space results. ***A***, Entrainment phase. Bar plots indicate coherence values for ROIs (mean ± standard error) in the left and right hemispheres. The entrainment effect (CONDITION–FREQUENCY interaction) was significant in all ROIs but higher in the right STG and EAC. ***B***, Target phase. Bar plots indicate power values for ROIs (mean ± standard error) in the left and right hemispheres. The entrainment effect (CONDITION–FREQUENCY interaction) was significant in all ROIs but higher in left EAC and FOP. For the complete list of the subareas in each ROI, please refer to Extended Data Table 3-1.

Source space analysis revealed a significant CONDITION–FREQUENCY interaction and a main effect of CONDITION in every ROI. Post hoc tests within every condition (0.6 vs 0.9 contrast) showed higher coherence at 0.6 Hz in the SLOW condition and at 0.9 Hz in the FAST condition (Table 1; Fig. 4*A*). These results show that brain synchronization to the pitch contour was significant in all ROIs. To further quantify differences in the entrainment effect between areas and hemispheres, we computed the rate-specific response (RSR) index, which reflects overall entrainment strength for every area (van Bree et al., 2021). Using this index as an outcome, we ran a new linear mixed-effects model with AREA, HEMISPHERE, and the AREA–HEMISPHERE interaction as predictors. Our model showed a significant AREA–HEMISPHERE interaction (*F* = 10.5, df = 3,245, *p* < 0.001) and the main effects for both AREA (*F* = 31.18, df = 3,245, *p* < 0.001) and HEMISPHERE (*F* = 72.68, df = 1,245, *p* < 0.001). Post hoc testing revealed that entrainment was stronger in the right EAC (*T* = 5.25, df = 245, *p* < 0.001) and STG (*T* = 2.83, df = 245, *p* < 0.001). These results are consistent with previous findings on prosodic tracking in right temporal regions (Bourguignon et al., 2013).

10.1523/JNEUROSCI.1041-23.2024.t3-1 Table 3-1 Areas, comprising regions of interest (ROIs). Download Table 3-1, DOCX file.

**Table 1.** Source space statistics for coherence in the entrainment phase

	FREQUENCY^a^		CONDITION^a^		FREQUENCY –CONDITION^a^		Post hoc *t* tests within a condition			
							SLOW		FAST	
	*F*	*p*	*F*	*p*	*F*	*p*	*T*	*p*	*T*	*p*
Left										
EAC	0.008	0.92	6.18	<0.05	68	<0.001	6	<0.001	−5.86	<0.001
STG	0.05	0.82	5.44	<0.05	69	<0.001	5.7	<0.001	−5.79	<0.001
IFG	2.41	0.12	5.54	<0.05	34	<0.001	3.57	0.001	−4.32	<0.001
FOP	1.75	0.18	10	<0.05	47	<0.001	4.6	<0.001	−5.17	<0.001
Right										
EAC	0.21	0.65	6.75	<0.05	163	<0.001	7.89	<0.001	−8.84	<0.001
STG	0.04	0.85	9.5	<0.05	203	<0.001	8.98	<0.001	−11	<0.001
IFG	2.16	0.15	10	<0.05	68	<0.001	5.42	<0.001	−5.85	<0.001
FOP	<0.001	0.98	9.4	<0.05	110	<0.001	7.93	<0.001	−6.48	<0.001

a Degrees of freedom for all reported LME models were equal to 1 (numerator) and 105 (denominator).

**Figure 5.**
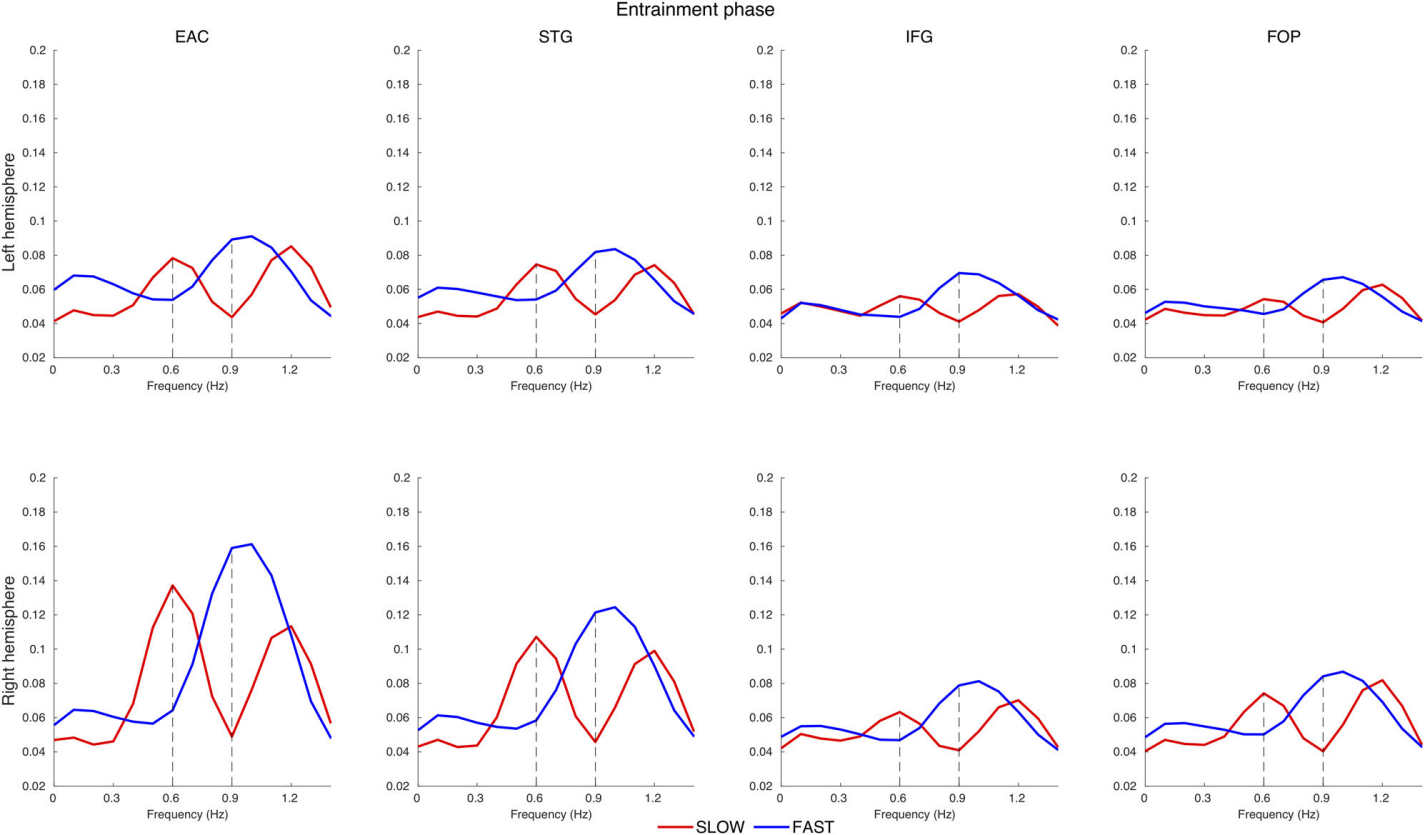
Coherence spectrum in the source space in the entrainment phase for different ROIs. Distinct peaks are visible: 0.9 Hz for the FAST condition and 0.6 and 1.2 (harmonics of 0.6) for the SLOW condition. These peaks correspond to the respective occurrence frequencies of the contours. The coherence values are higher in the right STG and EAC, in line with statistical analysis results. The *x*-axis is limited to 1.5 Hz for display purposes.

Visual inspection of the coherence spectrum in the corresponding ROIs (Fig. 5) confirms the coherence peaks at 0.6 and 1.2 Hz (harmonics of 0.6) for the SLOW condition and at 0.9 Hz for the FAST condition. The coherence values are higher in the right STG and EAC, which corresponds to our statistical analysis results.

### Delta-band activity carriers the contextual rhythm into the future

#### Sensor space – frequency analysis

During the target phase, sensor space statistics for the 0.6 versus 0.9 Hz contrast in SLOW revealed a significant positive cluster with a fronto-centro-occipital topography, shifted to the right (cluster-sum *t*_(34)_ = 66, cluster-level *p* < 0.001, corrected; peak at sensor MEG2641, peak-level *t*_(34)_ = 5.18, peak-level *p* < 0.001; Fig. 3*B*; for MEG sensors layout, see Extended Data Fig. 2-1). In turn, the 0.6 versus 0.9 Hz contrast in FAST exposed a negative cluster (cluster-sum *t*_(34)_ = −942, cluster-level *p* < 0.001, corrected; peak at sensor MEG2621, peak-level *t*_(34)_ = −18.58, peak-level *p* < 0.001). Finally, the comparison of difference maps (i.e., SLOW vs FAST) revealed a positive cluster (cluster-sum *t*_(34)_ = 706, cluster-level *p* < 0.001, corrected; peak at sensor MEG1731, peak-level *t*_(34)_ = 13.3, peak-level *p* < 0.001). The latter two clusters comprised all sensors. Peak sensors for SLOW and FAST clusters were located in the right temporal region, while the peak sensor for the difference cluster was located in the left parieto-occipital region. Taken together, sensor space results in the target phase strongly suggest that entrainment to prosody persists beyond stimulus offset.

#### Source space – frequency analysis

Source-level analyses showed a CONDITION–FREQUENCY interaction and a main effect of CONDITION for every ROI. Post hoc *t* tests for frequencies within every condition (0.6 vs 0.9 contrast) revealed that power was significantly higher at 0.6 Hz in SLOW and at 0.9 Hz in FAST (Table 2; Fig. 4*B*). Linear mixed-effects model for the differences between areas and hemispheres revealed a main effect of AREA (*F* = 38.82, df = 3,245, *p* < 0.001) and an AREA–HEMISPHERE interaction (*F* = 13.76, df = 3,245, *p* < 0.001). Post hoc testing showed that entrainment power was higher in the left EAC (*T* = 2.82, df = 245, *p* = 0.005) and left FOP (*T* = 4.52, df = 245, *p* < 0.001). This arrangement of source space activity is compatible with our hypothesis, as frontal regions are known to be associated with predictions and cognitive control (Alexander and Brown, 2018; Dürschmid et al., 2019). Our study further confirms evidence from the literature concerning a trend for the left lateralization of those functions (Jakuszeit et al., 2013; Matchin et al., 2017; Avenanti et al., 2018).

**Table 2.** Source space statistics for power in the target phase

	FREQUENCY^a^		CONDITION^a^		FREQUENCY–CONDITION^a^		Post hoc *t* tests within a condition			
							SLOW		FAST	
	*F*	*p*	*F*	*p*	*F*	*p*	*T*	*p*	*T*	*p*
Left										
EAC	0.94	0.34	482	<0.001	43	<0.001	11	<0.001	−12	<0.001
STG	0.84	0.36	535	<0.001	39	<0.001	15	<0.001	−12	<0.001
IFG	1.93	0.17	552	<0.001	44	<0.001	13	<0.001	−11	<0.001
FOP	1.14	0.34	501	<0.001	38	<0.001	14	<0.001	−11	<0.001
Right										
EAC	0.04	0.84	518	<0.001	35	<0.001	8.87	<0.001	−10	<0.001
STG	2.3	0.13	724	<0.001	51	<0.001	18	<0.001	−14	<0.001
IFG	1.99	0.16	449	<0.001	31	<0.001	12	<0.001	−14	<0.001
FOP	1	0.32	333	<0.001	24	<0.001	10	<0.001	−13	<0.001

a Degrees of freedom for all reported LME models were equal to 1 (numerator) and 105 (denominator).

**Figure 6.**
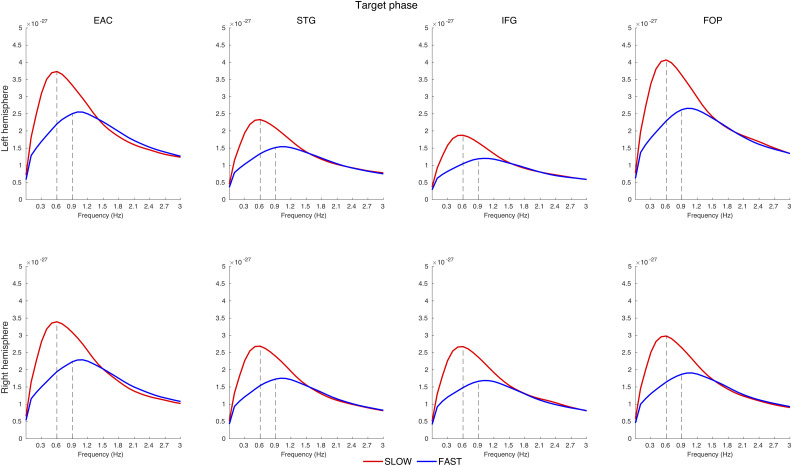
Power spectrum in the source space in the target phase for different ROIs. Distinct peaks are visible: 0.9 Hz for the FAST condition and 0.6 for the SLOW condition. These peaks correspond to the respective occurrence frequencies of the contours. The power values are higher in left FOP and EAC, in line with statistical analysis results.

Visual inspection of the power spectrum in the corresponding ROIs (Fig. 6) confirms the power peaks at 0.6 Hz for the SLOW condition and at 0.9 Hz for the FAST condition. The power values are higher in left FOP and EAC, which corresponds to our statistical analysis results.

### Falsified predictions are associated with an omission effect

#### Sensor space–ERF analysis

**Figure 7.**
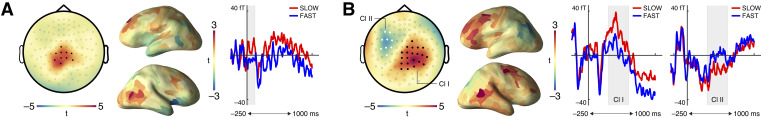
ERF analysis. ***A***, Earlier window (0–120 ms). Left: Topographical distribution of the ERF effect; cluster with significant differences highlighted. Right: Grand average ERF wave across the cluster. SLOW condition is associated with increased ERF amplitude at the onset of missing verbs in SHORT sentences relative to the FAST condition. Time window of interest (0–120 ms) marked in gray. Middle: Source space. The stronger red color indicates areas where the SLOW condition induced higher activation than FAST (Table 5), while the reverse is indicated by the stronger blue color. ***B***, Later window (300–600 ms). Left: Topographical distribution of omission ERF effect; clusters with significant differences highlighted (positive cluster, black; negative cluster, white). Right: Grand average ERF waves across the clusters of sensors (left, positive cluster; right, negative cluster). For both clusters, the SLOW condition is associated with increased ERF amplitude at the onset of missing verbs in SHORT sentences relative to the FAST condition. Time window of interest (300–600 ms) marked in gray. Middle, Source space. The stronger red color indicates areas where the SLOW condition induced higher activation than FAST (Table 3), while the reverse is indicated by the stronger blue color.

The ERF analysis showed a significant positive right centroparietal cluster (cluster-sum *t*_(34)_ = 23, cluster-level *p* < 0.001, corrected; peak at sensor MEG2231, peak-level *t*_(34)_ = 5.29, peak-level *p* < 0.001, corrected) and a significant neighboring negative cluster (cluster-sum *t*_(34)_ = −7.9, cluster-level *p* = 0.019, corrected; peak at sensor MEG0221, peak-level *t*_(34)_ = −3.57, peak-level *p* = 0.001, corrected; Fig. 7*B*, left; for MEG sensors layout, see Extended Data Fig. 2-1). Note that the polarity of the cluster in ERF analysis is not informative about the direction of the effect. In both clusters, the SLOW condition induced higher-amplitude ERF waveforms than the FAST condition (Fig. 7*B*, right). Therefore, we can infer that participants did indeed predict longer sentences under the SLOW entrainment and the incongruent condition caused an omission ERF effect.

#### Source space–ERF analysis

Source space analysis revealed multiple areas where the activation for the SLOW condition was significantly different (uncorrected) from the FAST condition; we here report 10 regions with maximum differences in the later time window (Table 3). The areas where the activation for the SLOW condition was significantly higher than for the FAST condition (positive *T*-values) include the temporo-parieto-occipital junction and a number of frontal and premotor regions (Fig. 7*B*, middle). Overall, the pattern of source-level activity suggests that entrainment originates in the right temporal cortices and is maintained in the left frontal cortex, which generates temporal predictions; in case those predictions are incongruent, an M300 ERF emerges from a variety of cortical generators.

**Table 3.** Regions with significant differences in the ERF 300–600 ms contrast (source space)

	*T*	df	*p*
Left			
Area i6–8 (dorsolateral prefrontal cortex)	2.87	34	<0.05
Area 9m (medial prefrontal cortex)	2.67	34	<0.05
Area IFSa (inferior frontal cortex)	2.37	34	<0.05
Area p24 (medial prefrontal cortex)	2.32	34	<0.05
Area 47l (inferior frontal cortex)	2.29	34	<0.05
Area 8C (dorsolateral prefrontal cortex)	2.3	34	<0.05
Area PGs (inferior parietal cortex)	−2.22	34	<0.05
Right			
Temporo-parieto-occipital junction (Area 2)	3.07	34	<0.05
Frontal eye field	2.98	34	<0.05
Area i6–8 (dorsolateral prefrontal cortex)	2.48	34	<0.05

### Additional analyses

#### Source space – frequency analysis

**Figure 8.**
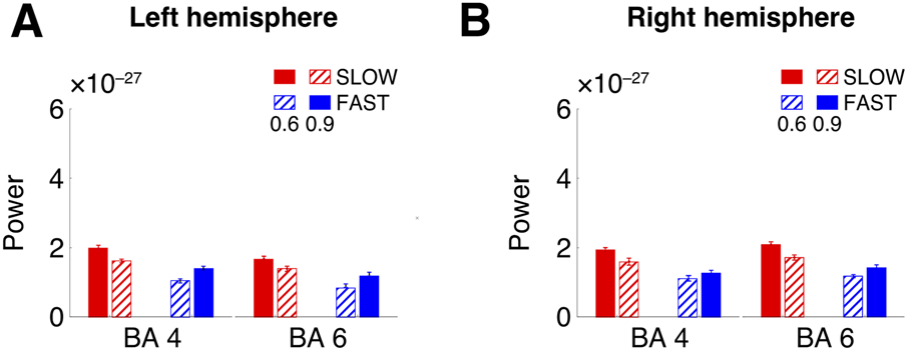
Source space results—entrainment in the motor cortex in the target phase. Bar plots indicate power values (mean ± standard error) in the left and right hemispheres. The entrainment effect (CONDITION–FREQUENCY interaction) was significant in both motor and premotor regions (Brodmann areas 4 and 6).

In addition to temporal prediction processing being associated with IFG and FOP, previous literature has also demonstrated the potential involvement of the motor cortex in temporal predictions. In particular, evidence suggests that temporal predictions in the motor cortex, subserved by beta-band oscillations, can upregulate auditory–motor interaction via a top-down phase reset of the oscillations in the auditory cortex (Morillon and Baillet, 2017; Keitel et al., 2018; Assaneo et al., 2021). These oscillations are thought to function as an endogenous temporal constraint, facilitating bottom-up processing (Rimmele et al., 2018). Furthermore, in the current paradigm, predictive processing as a stage of decision-making in order to answer the comprehension question could potentially involve motor preparation. In order to investigate these hypotheses, we conducted a control analysis of the entrainment effect in premotor (Brodmann area 6) and motor (Brodmann area 4) areas (for the list of the atlas subareas, please refer to Extended Data Table 3-1). The results demonstrated significant entrainment in both areas and hemispheres (Fig. 8).

Statistical analyses showed a CONDITION–FREQUENCY interaction and a main effect of CONDITION for every ROI (Table 4). Post hoc *t* tests for frequencies within every condition (0.6 vs 0.9 contrast) also confirmed that power was significantly higher at 0.6 Hz in SLOW and at 0.9 Hz in FAST, explaining the interaction effect.

**Table 4.** Source space statistics for power in the target phase (motor and premotor areas)

	FREQUENCY^a^		CONDITION^a^		FREQUENCY–CONDITION^a^		Post hoc *t* tests within a condition			
							SLOW		FAST	
	*F*	*p*	*F*	*p*	*F*	*p*	*T*	*p*	*T*	*p*
Left										
BA4	4.48	<0.05	732	<0.001	62	<0.001	16.8	<0.001	−11.57	<0.001
BA6	5.77	<0.05	751	<0.001	64	<0.001	14.19	<0.001	−12.07	<0.001
Right										
BA4	4.26	<0.05	467	<0.001	41	<0.001	13.05	<0.001	−13.65	<0.001
BA6	3.03	0.085	453	<0.001	37	<0.001	12.86	<0.001	−13.5	<0.001

a Degrees of freedom for all reported LME models were equal to 1 (numerator) and 105 (denominator).

#### ERF analysis

In addition to later effects, prosodic processing has also been demonstrated at very early stages (Tomasello et al., 2022). In order to investigate whether those effects persist in the target phase of prosodic entrainment, we conducted an additional ERF analysis in the time window of 0–120 ms after the critical word onset on the same data as used for the M300 analysis. The results revealed significant differences in a central positive cluster, with higher activation for the SLOW condition (Fig. 7*A* left). This effect can be interpreted as an early omission response, akin to prosody expectation violation (Paulmann et al., 2012; Kakouros et al., 2018) induced by prosodic entrainment.

We further attempted a source reconstruction on the early ERF response (Fig. 7*A*, middle). We report 10 regions where the ERF difference between the SLOW and FAST conditions was maximal (uncorrected; Table 5). The pattern appears quite similar to the source pattern obtained for the 300–600 ms omission ERF (Fig. 7*B*, middle), with higher activation for the SLOW condition in frontal areas on the left and temporo-parieto-occipital junction (TPOJ) on the right. The TPOJ activation could potentially be related to visual omission effects (the omitted word was expected to be presented in a visual mode). However, we here need to note that source reconstruction of ERF with beamforming can potentially be problematic, since this approach underestimates highly covariant sources, and does not consider the degree to which estimated source signals explain observed sensor signals (Kuznetsova et al., 2021; O’Reilly et al., 2023). The current results, therefore, should be treated with caution.

**Table 5.** Regions with significant differences in the ERF 0–120 ms contrast (source space)

	*T*	df	*p*
Left			
Area 9p (dorsolateral prefrontal cortex)	2.73	34	<0.05
Area PeEc (medial temporal cortex)	−2.38	34	<0.05
Area 8BL (dorsolateral prefrontal cortex)	2.2	34	<0.05
Area 8BM (medial prefrontal cortex)	2.13	34	<0.05
Right			
Middle temporal cortex	2.88	34	<0.05
Area AAIC (anterior insular cortex)	−2.71	34	<0.05
Area Pir (anterior insular cortex)	−2.61	34	<0.05
Area V7 (visual cortex)	−2.22	34	<0.05
Temporo-parieto-occipital junction (Area 2)	2.11	34	<0.05
Posterior orbitofrontal complex	−1.98	34	<0.05

## Discussion

Our results suggest that speech prosody subserves temporal predictions in language via electrophysiological inheritance of contextual temporal patterns. This converges with recent evidence for similar mechanisms supporting temporal predictions in the domain of auditory perception more generally (Stefanics et al., 2010; Breska and Deouell, 2017; Herbst et al., 2022). The facilitatory effect of delta-band entrainment on nonlinguistic auditory perception (e.g., tone sequences) has been shown earlier (Henry and Obleser, 2012; Hickok et al., 2015). While in the preceding studies, the effect was demonstrated for auditory tones, we here report delta-band entrainment in the context of language processing. To our knowledge, earlier reports of behaviorally relevant sustained linguistic entrainment have been published only for higher frequencies (Kösem et al., 2018; van Bree et al., 2021). The setup of the current study allows us to adopt a strong interpretation in terms of entrainment proper (Rimmele et al., 2018; Obleser and Kayser, 2019; L. Meyer et al., 2020a,b)—that is, electrophysiological neural activity does not just mimic stimulation frequencies at the time of stimulation, but maintains these for a subsequent period (Stefanics et al., 2010; Kösem et al., 2018; van Bree et al., 2021).

The present findings stress the necessity of incorporating mechanisms of temporal prediction into current neurobiological, psycholinguistic, and computational models of human speech and language (Friederici and Alter, 2004; Hickok and Poeppel, 2007; Alexander and Brown, 2018; Ten Oever and Martin, 2021). Our source space results are consistent with the notion that such temporal predictions operationalize auditory–motor interaction (Keitel et al., 2018; Assaneo et al., 2021; Kern et al., 2021; Lubinus et al., 2023). Recent studies have proposed models where temporal predictions operate through top-down phase resetting of electrophysiological activity mediated by the motor system or higher-order language—or attention-related systems (for a review, see Rimmele et al., 2018). This mechanism facilitates bottom-up perceptual processing, while intrinsic temporal characteristics of neural activity might serve as intrinsic temporal constraints. Furthermore, motor cortices have also been related to predictive processing on a broader scale (Grisoni et al., 2017, 2021). Interestingly, it has been shown that their engagement is dependent on the specific nature of the predictions being made—for instance, frontocentral-sensorimotor activation for tool-related words (Grisoni et al., 2021), dorsolateral hand motor activation for hand-related words (e.g., “write”), and ventral motor activation for face-related words (“talk”; Grisoni et al., 2017). Our additional analysis confirmed a sustained entrainment effect in the motor cortices.

Moreover, studies on lateralization of predictive processes indicate that bottom-up auditory processing is primarily associated with auditory cortices in the right hemisphere, while top-down control tends to be left-lateralized (Keitel et al., 2018; Assaneo et al., 2019). This is in line with our results. In the entrainment phase, right STG and EAC showed significantly higher coherence with the pitch contour. These findings are also consistent with the assumed neural substrate of prosody (M. Meyer et al., 2004; Bourguignon et al., 2013; Sammler et al., 2015). In the target phase, however, activity at the conditioning frequency moved to the left frontal cortices, involved in temporal prediction in language (Jakuszeit et al., 2013; Matchin et al., 2017). Left FOP activity is also generally known to correspond to syntactic processing or prediction (Friederici et al., 2003, 2006; Bubic et al., 2009), which in our paradigm is engaged by prior entrainment. Sustained entrainment in frontal areas could also potentially reflect the broader engagement of the frontal cortex in decision-making regarding the experimental task. Temporal prediction could be the first and integral stage of decision-making processing, facilitating the upcoming sentence analysis and, therefore, forming preliminary answers to the comprehension question.

Our interpretation of the omission response in terms of a temporal prediction error is in line with previous reports from auditory perception (Stefanics et al., 2010; Herbst et al., 2022), where temporal predictions carried by delta-band activity modulated the amplitude of the P300 component on target tones. The current ERF effect is likely an omitted stimulus potential, bearing characteristics of both P300 and P600 components (or M300/M600, as their MEG counterparts; Bullock et al., 1994; Karamürsel and Bullock, 2000; Penney, 2004; Nakano et al., 2014; Ragazzoni et al., 2019). This interpretation conforms to the idea that the P600 component in the language domain is a member of the greater P300 family (Sassenhagen and Fiebach, 2019). The widely distributed array of underlying cortical sources observed here is in line with prior reconstructions of the P300, which include diverse distributed generators (Brázdil et al., 2005; Linden, 2005; Bocquillon et al., 2011; Ragazzoni et al., 2019). The temporo-parieto-occipital junction as a generator of P300 was previously reported in multiple deviating stimuli studies, together with dorsolateral prefrontal areas (Daffner et al., 2003; Volpe et al., 2007; Strobel et al., 2008). Moreover, a study on semantic predictions has also previously reported temporo-parieto-occipital activation (Grisoni et al., 2021). A visual oddball study (Bledowski et al., 2004) also reports activation in the frontal eye field, similar to our results. These findings are in line with our conclusions, since, in our paradigm, the stimulation in the target phase was in the visual modality. Moreover, multiple omitted-target studies also find activation in the inferior frontal cortex (Bledowski et al., 2004; Volpe et al., 2007; Ragazzoni et al., 2019), which is corroborated by our results. This evidence further strengthens our claim of the left frontal cortex involvement in predictive processing (Jakuszeit et al., 2013; Matchin et al., 2017).

Our complementary analysis also revealed a significant earlier omission response in the time window of 0–120 ms. This could be potentially related to prosodic entrainment inducing early omission effects, similar to prosody expectation violations (Paulmann et al., 2012; Kakouros et al., 2018; Tomasello et al., 2022). The source localization pattern of this earlier ERF resembles that of the M300/M600 omission effect; however, it is important to note that source localization procedures for ERFs should be regarded with caution (Kuznetsova et al., 2021; O’Reilly et al., 2023).

The current study bears important implications for the general conceptualization of the neurobiological underpinnings of interpersonal communication. Prosodic entrainment could potentially be used as a facilitatory mechanism in dialog, enhancing mutual comprehension (Edlund, 2011; Breen et al., 2016b; Lehnert-LeHouillier et al., 2020). In line with the implicit prosody account (Fodor, 2002; Breen, 2014; Perrone-Bertolotti et al., 2014), in the current experiment, based on prior prosodic entrainment listeners would silently generate the same prosodic structures during the reading of subsequent sentences, which would influence their temporal prediction regarding the length of the sentence. This mechanism can be applied to communication in a dialog: the listener could potentially entrain to the speaker's prosody, generating silent prosodic structures and forming temporal predictions as to what the speaker is going to say next.

These findings could be incorporated into broader models of alignment in dialog (Pickering and Garrod, 2004, 2021). This account postulates that interlocutors in a dialog utilize linguistic representations that automatically align on many levels, facilitating production and comprehension processes. In principle, this general mechanism could operate over various acoustic features, including rate, intensity, voice quality, and pitch (Brennan, 1996; Teoh et al., 2019). These features need to exhibit sufficient rhythmicity to facilitate conversational entrainment. Although ubiquitous rhythmicity is not characteristic of human speech, previous research has demonstrated that speech is rhythmic enough on the prosodic level to trigger entrainment in dialog (Inbar et al., 2020; Stehwien and Meyer, 2021); furthermore, rhythmic intonational units in speech evoke a neural response (Inbar et al., 2023). Further research quantifying the rhythmicity of naturalistic speech and investigating the differences in prosodic entrainment for rhythmic and nonrhythmic parts could provide additional evidence for this claim.

Work on interpersonal communication also supports the notion that prosody is involved in conversational entrainment (Levitan and Hirschberg, 2011; Levitan et al., 2012; Reichel et al., 2018). It has previously been demonstrated that prosody conveys communicative intentions in speech irrespective of emotion (Hellbernd and Sammler, 2016). Neurobiologically these effects have been localized to the auditory ventral stream and articulatory–motor regions (Hellbernd and Sammler, 2018; Tomasello et al., 2022). On a more general level, it has been proposed that human brains synchronize communicative activities through an oscillatory signal (Wilson and Wilson, 2005; Hasson et al., 2012). Furthermore, it has not only been found that the listener's brain activity is spatially and temporally coupled with the speaker's but that the listener's frontal areas also exhibit predictive anticipatory responses during dialog (Stephens et al., 2010). Our present results, showing prosodic entrainment as a mechanism of temporal predictions in listening comprehension, could therefore extend to scenarios in dialog. Altogether, prosodic entrainment appears to play an important role in enhancing communication processes via temporal prediction.

The focus of the current study has been the investigation of purely prosodic entrainment. However, it is pertinent to note that potentially similar effects could be evoked by presenting nonprosodic stimuli of fixed rhythmic duration (e.g., acoustic stimuli with a flat pitch or visual stimulation). While the timing of the experiment and the necessity of a substantial number of trials did not permit us to include a control nonprosodic condition in the current paradigm, future work would be beneficial to distinguish between prosodic versus merely acoustic/visual entrainment effects.
