# Supplementary figures and images for: Speech Prosody Serves Temporal Prediction of Language via Contextual Entrainment
Source: J Neurosci. 2024 Jun 5;44(28):e1041232024. doi: 10.1523/JNEUROSCI.1041-23.2024 (PMC11236583; doi:10.1523/JNEUROSCI.1041-23.2024)

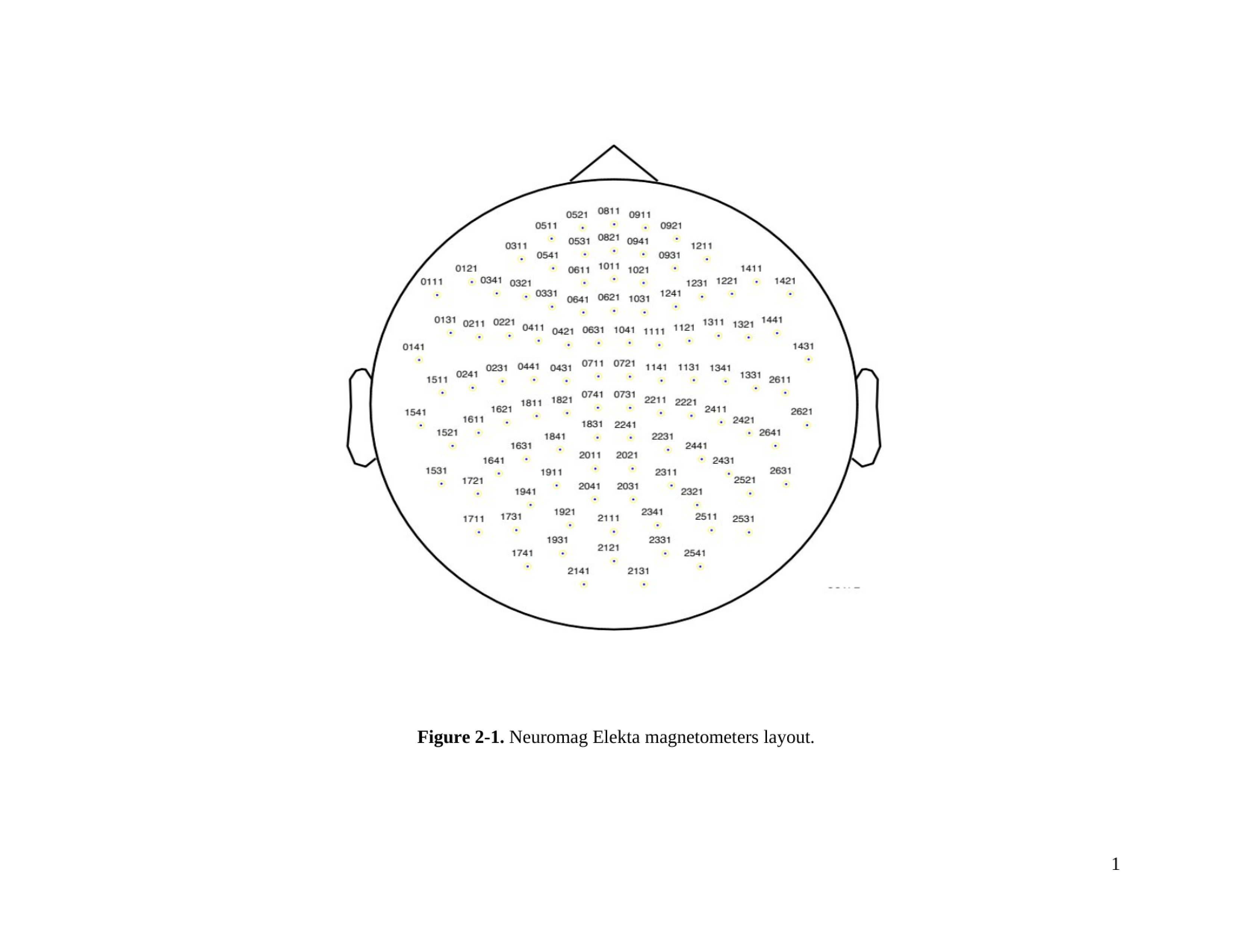

Supplement: Figure 2-1 — Download Figure 2-1, TIF file. [file jneuro-44-e1041232024-s002.tif]
