# Supplementary material for: Speech Prosody Serves Temporal Prediction of Language via Contextual Entrainment
Source: J Neurosci. 2024 Jun 5;44(28):e1041232024. doi: 10.1523/JNEUROSCI.1041-23.2024 (PMC11236583; doi:10.1523/JNEUROSCI.1041-23.2024)
Supplement: Table 3-1 — Areas, comprising regions of interest (ROIs). Download Table 3-1, DOCX file. [file jneuro-44-e1041232024-s001.docx]

**Table 3-1. Areas, comprising regions of interest (ROIs).**

Region and area labels are provided according to (Glasser et al., 2016) atlas.

1. *Early Auditory Cortex (EAC, region 10)*

- Primary auditory cortex ('A1')
- Lateral Belt (‘LBelt’),
- Medial Belt (‘MBelt’)
- Para-Belt (‘PBelt’)
- Retro-insular cortex (‘RI’)

1. *Superior Temporal Gyrus (STG, region 11)*

- Area A4 ('A4’)
- Area A5(‘A5’)
- Anterior superior temporal area(‘TA2’)
- Anterior superior temporal gyrus (‘STGa’)
- Dorsal anterior superior temporal sulcus (‘STSda’)
- Dorsal posterior superior temporal sulcus (‘STSdp’)
- Ventral anterior superior temporal sulcus (‘STSva’)
- Ventral posterior superior temporal sulcus (‘STSvp’)

1. *Inferior frontal gyrus (IFG, region 21)*

- Area 44 (‘44’)
- Area 45 (‘45’)

1. *Frontal Opercular Cortex (FOP region 12)*

- Frontal opercular area 2 (‘FOP2’)
- Frontal opercular area 3 (‘FOP3’)
- Frontal opercular area 4 (‘FOP4’)
- Frontal opercular area 5 (‘FOP5’)
- Anterior ventral insular area (‘AVI’)

1. *(additional analysis) Motor cortex (MC, region 6)*

- Primary motor cortex, BA 4 (‘4’)

1. *(additional analysis) Premotor cortex – BA6 (PMC, region 8)*

- Anterior area 6 (‘6a’)
- Dorsal area 6 (‘6d’)
- Ventral area 6 (‘6v’)
- Rostral area 6 (‘6r’)
- Frontal eye field (‘FEF’)
- Premotor eye field (‘PEF’)
